# Thermoresponsive Polyoxazolines as Vectors for Transfection of Nucleic Acids

**DOI:** 10.3390/polym12112609

**Published:** 2020-11-06

**Authors:** Emi Haladjova, Stanislav Rangelov, Christo Tsvetanov

**Affiliations:** Institute of Polymers, Bulgarian Academy of Sciences, Acad. G. Bonchev St. 103-A, 1113 Sofia, Bulgaria; ehaladjova@polymer.bas.bg (E.H.); chtsvet@polymer.bas.bg (C.T.)

**Keywords:** Poly(2-oxazoline)s, polyethyleneimine, non-viral vectors, gene delivery, mesoglobules

## Abstract

Poly(2-oxazoline)s (POx) are an attractive platform for the development of non-viral gene delivery systems. The combination of POx moieties, exhibiting excellent biocompatibility, with DNA-binding polyethyleneimine (PEI) moieties into a single copolymer chain is a promising approach to balance toxicity and transfection efficiency. The versatility of POx in terms of type of substituent, copolymer composition, degree of polymerization, degree of hydrolysis, and chain architecture, as well as the introduction of stimuli-responsive properties, provides opportunities to finely tune the copolymer characteristics and physicochemical properties of the polyplexes to increase the biological performance. An overview of the current state of research in the POx–PEI-based gene delivery systems focusing particularly on thermosensitive POx is presented in this paper.

## 1. Introduction

Gene therapy refers to the treatment of acquired and hereditary genetic diseases by modifying and repairing the genetic structure [1,2,3]. It consists of the exogenous delivery of foreign therapeutic genetic material into target cells to add, replace, or edit a gene that is absent or abnormal and responsible for a disease. Despite the positive results produced by the engineered viruses, which are the first gene delivery agents, the viral therapies are associated with the high cost of treatment and low scalability as well as with a number of potential risks for the patients, as severe side effects have been made strikingly evident [4,5,6,7].

In the last few decades, non-viral gene delivery has been a focus of intensive research due to the opportunities it offers for safer and low-cost treatment [8,9,10,11,12,13], batch to batch reproducibility of materials, and access to large-scale production. The class of non-viral vectors includes different carriers based on inorganic particles, lipids (lipoplexes) [14,15], polymers (polyplexes) [12,13,14,15,16], surfactant and polymer micelles (micelleplexes) [17,18] as well as hybrid, e.g., lipid–polymer structures (lipopolyplexes) [19,20]. Among them, polymer-based vectors have been found to be advantageous, since they could address many of the gene delivery issues. The contemporary polymer chemistry affords synthetic versatility to the target design of vectors with specific properties including the tailorability of the systems, flexibility to formulation design, amenability to modifications, and targeting of specific cells [10,11,12,15]. The main synthetic polymer that is used extensively to prepare polyplexes is polyethyleneimine (PEI) [21,22,23]. This polymer is commercially available in both linear and branched chain topology and different molecular weights. PEI effectively binds and condenses DNA to nanoscale polyplex particles and exhibits an ability to escape the endolysosomal compartments due to the so-called *proton sponge* effect [9,24]. It is often considered to be the *gold standard* for the polymer-based vectors for DNA delivery [17,18], although its relatively high toxicity [25,26] is an issue.

While branched PEI (BPEI) is normally synthesized via the polymerization of aziridine, linear PEI (LPEI) is obtained by the hydrolysis of poly(2-oxazoline)s (POx) [27,28,29,30,31,32]. POx are a class of polymers that have received significant attention in the past decades [27,28,29,30,31,32] and are currently discussed as an upcoming polymer platform for various biomedical applications. The POx family represents a large variety of polymers with chemical and structural versatility and functionalities. They are accessible by the living cationic ring opening polymerization of 2-oxazolines providing good control over the polymerization reaction, resulting in a wide variety of well-defined polymers [27,28]. An important feature of POx is the unique property of some family members to undergo a reversible soluble-to-insoluble state transition in response to small changes in temperature [28,33] and exhibit a lower critical solution temperature (LCST) in water. This thermoresponsive behavior assigns them as smart materials and further brings about a broad spectrum of specific properties. The structural relation to natural polypeptides gives them prominent biocompatibility fully competing the well-known polyethylene glycol (PEG) [27,28,34]. Furthermore, they have been reported to provide also *stealth* properties to improve pharmacogenetics and pharmacokinetics and to exhibit rapid clearance from the human body [27,28]. The introduction of side-chain functionalities is another important advantage of POx over PEG, which is chemically inert and suffers from a lack of functional groups along the polymer backbone. Apparently, POx represent an attractive platform for the development of various biomaterials, especially for drug and gene delivery [30]. 

Being a convenient source for obtaining LPEI by acidic or basic hydrolysis **[31,32]**, POx have been of interest in the context of gene delivery. Furthermore, the partial hydrolysis is highly desirable, as it leads to copolymers consisting of two different moieties—(i) cationic, pH-responsive, and DNA binding and (ii) non-ionic, water-soluble or exhibiting LCST properties, and biocompatible. Although this combination of properties seems to be promising and attractive for the development of non-viral vectors, the POx–PEI copolymers are somewhat under-researched. The aim of this paper is to overview the current state of research in the POx–PEI-based gene delivery systems focusing particularly on thermosensitive POx. We try to relate the physicochemical characteristics, aqueous solution and LCST properties to biological performance and effectiveness as gene delivery vectors of POx–PEI copolymers. Examples for POx–PEI copolymers derived from POx, which are not thermosensitive, are also given to complement the picture and for better understanding of their behavior. 

## 2. Aqueous Solution Behavior of POx–PEI Copolymers

The anionic nature of DNA is exploited to drive complexation via electrostatic interactions with oppositely charged (cationic) polymers or polymers bearing cationic moieties [12,13]. Polyplexes are typically formed simply by mixing aqueous solutions of molecularly dissolved DNA and polymer at a given amino-to-phosphate groups (N/P) ratio. The aqueous solution properties of thermosensitive POx and their copolymers with PEI can be distinctively different depending on temperature, which, in turn, can affect the physicochemical characteristics, structure, and properties and, possibly, the entire biological performance and transfection effectiveness of the resulting polyplexes. Therefore, knowledge and understanding of the aqueous solution properties of the copolymers is of key importance.

**Figure 1:**
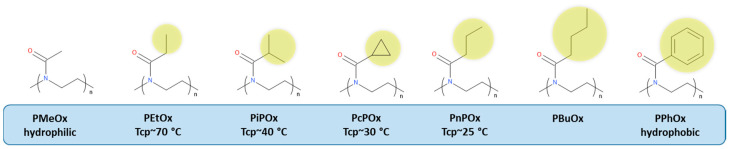
Chemical structure and water solubility of poly(2-oxazoline)s (POx) as a function of alkyl side chain.

The water solubility of POx strongly depends on the 2-substituent (Figure 1). Thus, poly(2-methyl-2-oxazoline) (PMeOx) is fully hydrophilic, exhibiting excellent water solubility in the whole temperature range from 0 to 100 °C. The ethyl side chain of poly(2-ethyl-2-oxazoline) (PEtOx) gives rise to thermosensitive properties with LCST in the range of 60–70 °C depending on the polymer molar mass, concentration, and addition of salts. Increasing the alkyl side chain by an extra carbon leads to a further decrease in the LCST. Although poly(iso-propyl-2-oxazoline) (PiPOx), poly(2-cyclopropyl-2-oxazoline) (PcPOx), and poly(n-propyl-2-oxazoline) (PnPOx) are structural isomers, their LCSTs differ (≈40 °C, ≈30 °C, and ≈25 °C, respectively). Poly(2-butyl-2-oxazoline) (PBuOx) is the first insoluble member of this family, and the further increase of the side chain produces entirely hydrophobic polymers. On the other hand, PEI is water-soluble, but its solubility increases upon heating, which has been attributed to the weakening of intra- and interchain hydrogen bonding between water and the amino groups of PEI [35,36,37]. Thus, the introduction of PEI moieties to the POx chain may have from a negligible to strong influence on the copolymer aqueous solution properties, depending on the 2-substituent, degree of polymerization (DP), degree of hydrolysis (DH), and temperature.

The partial hydrolysis does not substantially influence the good water solubility of PMeOx, as recently shown for a series of PMeOx–PEI copolymers differing in DP and DH [38]. These copolymers exhibited positive ζ potential in the range of 11 to 41 mV strongly depending on the DH: the higher the polycation content, the more positive the ζ potential. Furthermore, at the same DH, the ζ potential shifted to more positive values for the copolymers of higher DP. The authors explained this effect with differences in the charge density being larger for longer polymers due to more compact polymer coil [38].

**Figure 2:**
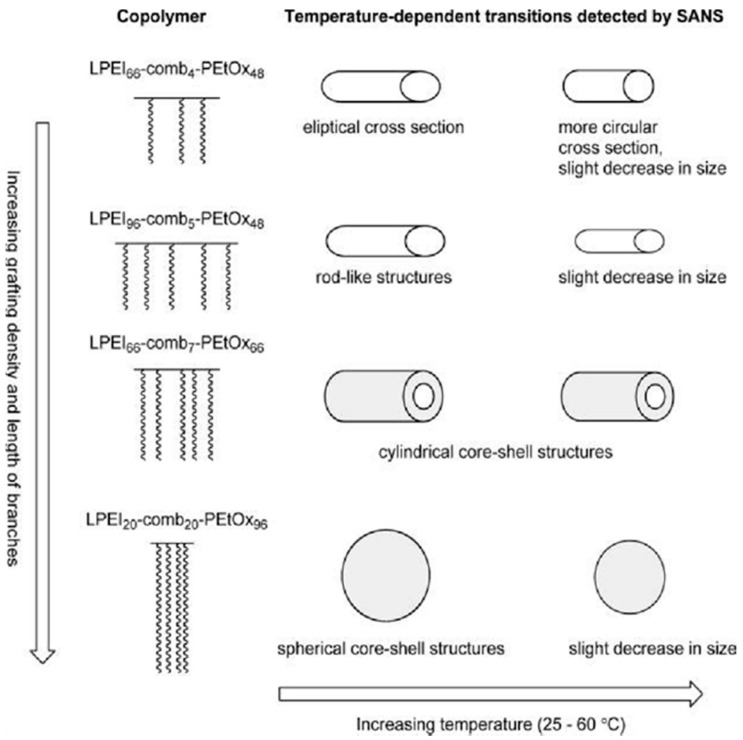
Aqueous solution behavior of poly(2-ethyl-2-oxazoline (PEtOx)–polyethyleneimine (PEI) comb-like copolymers and their shape transitions with temperature and composition determined by SAXS. Adapted from Halacheva et al. as published by the American Chemical Society [39].

It is noteworthy that for the POx–PEI copolymers composed of POx exhibiting LCST properties, the interactions of the two moieties with water change in opposite manners upon heating. A detailed study of the effect of temperature and polymer composition on the water solubility of comb-like PEtOx–PEI copolymers is presented by Halacheva et al. [39]. The aqueous solution properties of well-defined copolymers of varying grafting densities and the degree of polymerization of both PEI main chain and PEtOx graft chains were investigated by dynamic light scattering (DLS) and small angle X-ray scattering (SAXS). It was shown that these copolymers formed nanosized aggregates at low temperature with a hydrodynamic radius (R_h_) in the range of 110–156 nm. They were composed of compact LPEI domains surrounded by a thin hydrated PEtOx corona. The shape transitions of the copolymer aggregates with temperature and composition were observed (Figure 2). Increasing temperature led not only to particle compaction but also to their structural rearrangement [39,40]. As a consequence of the migration of PEI moieties toward the periphery of the particles, their ζ potential shifted to more positive values and even turned from negative to positive [40].

**Figure 3:**
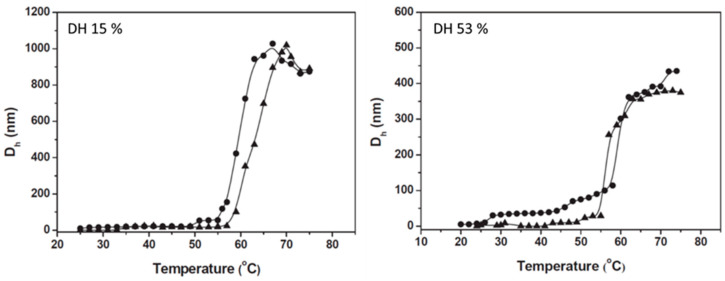
Variations of hydrodynamic diameters (D_h_) with temperature for aqueous dispersions of PiPOx–PEI copolymers of degrees of hydrolysis of 15% (**left**) and 53% (**right**) and concentrations of 0.05 mg/mL (triangles) and 0.1 mg/mL (circles). Phase transition temperatures are determined as the onset of aggregation. Adapted from Toncheva-Moncheva et al. as published by the Elsevier [42].

In many aspects, the behavior of poly(propyl-2-oxazoline)-polyethyleneimine (PPOx–PEI) copolymers is similar to that of PEtOx–PEI with more pronounced differences at lower and elevated temperatures [41,42]. PnPOx exhibits LCST properties with phase transition temperature ≈25 °C (Figure 1). Table 1 shows that upon the introduction of 5% amine functionality, the phase transition temperature in different media shifts to higher values and further increases with increasing PEI content [41]. In contrast, for PiPOx–PEI copolymers, the DH does not influence substantially the phase transition temperature, as shown elsewhere [42] (Figure 3).

**Figure 4:**
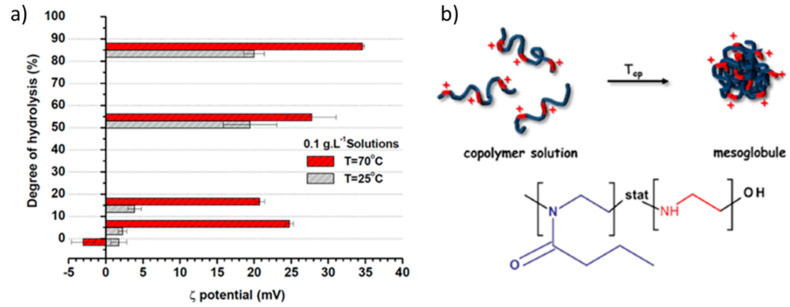
Variations of ζ potential of PiPOx–PEI copolymers with DH and temperature (**a**). Adapted from Toncheva-Moncheva et al. as published by the Elsevier [42]. Schematic representation of the formation of positively charged mesoglobules from PnPOx–PEI copolymers (**b**). Adapted from Mees et al. as published by the American Chemical Society [41].

Both PnPOx–PEI and PiPOx–PEI copolymers of low DH (up to 20%) exhibited nearly neutral ζ potential below the LCST (Table 2 and Figure 4a) [41,42,43], which was attributed not only to the low PEI content but also to the specific arrangement of macromolecules in which the ethylene imine units were shielded by the PPOx moieties. DOSY NMR and DLS analyses, performed above the LCST, revealed the formation of nano-sized particles (mesoglobules), as schematically depicted in Figure 4b [41]. The shift to strongly positive ζ potential (Table 2 and Figure 4a) was explained by structural rearrangements accompanied by relocation of the positive charges to the outer mesoglobules’ surface. Even for the copolymers of higher DH (above 50%), the ζ potential was found to shift to more positive values (Figure 4a), implying that the same events of structural rearrangement upon heating above the LCST took place. It is noteworthy that the introduction of ethylene imine units has been found to completely inhibit the high-temperature crystallization of PiPOx [42]. The latter is a specific property observed exclusively for PiPOx, which has been rationalized in terms of the facilitation of dipolar interactions between amide groups upon long-term annealing at temperatures above the LCST leading to an irreversible formation of micron-sized coagulated particles built of nanofibers [44]. It could be hypothesized here that the observed formation of positively charged mesoglobules resulting from the thermosensitivity of POx is beneficial, since such structures would increase the accessibility of the positive charges for complexation with DNA.

**Figure 5:**
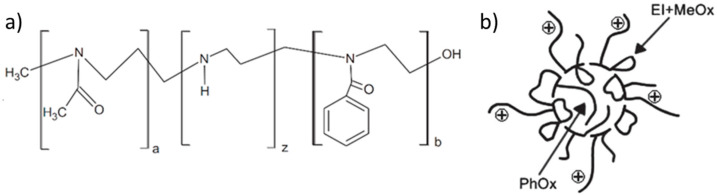
Chemical structure of poly(2-methyl-2-oxazoline)-polyethyleneimine-poly(2-phenyl-2-oxazoline) (PMeOx–PEI–PPhOx) gradient copolymers (**a**) and schematic representation of the formed aggregates (**b**). Adapted from Vlassi et al. as published by the Wiley-VCH Verlag GmbH [45].

Vlassi and Pispas [45] reported on formation of polymer aggregates based on poly(2-methyl-2-oxazoline)-polyethyleneimine-poly(2-phenyl-2-oxazoline) (PMeOx–PEI–PPhOx) gradient copolymers (Figure 5a). The copolymers were amphiphilic but, noteworthily, not thermosensitive, and formed aggregates composed of a PPhOx core surrounded by randomly situated PMeOx segments and PEI loops. The aggregates exhibited entirely positive ζ potential (24–35 mV) and size ranging from 95 to 164 nm. The proposed structure of the aggregates (Figure 5b) is quite similar to those formed by the thermoresponsive POx–PEI copolymers at elevated temperatures.

## 3. Biocompatibility of POx–PEI Copolymers

The biocompatibility of POx has been widely investigated and frequently reviewed [27,28,30]. Numerous studies indicate an excellent compatibility of POx with various biological systems. The combination of POx with PEI is undoubtedly a good strategy for suppressing the extremely high polycation cytotoxicity, which, as noted above, is an issue in gene delivery. In general, studies have shown that POx–PEI copolymers are characterized by significantly lower toxicity compared to PEI alone. Below, we outline some basic dependencies regarding the copolymer characteristics.

**Figure 6:**
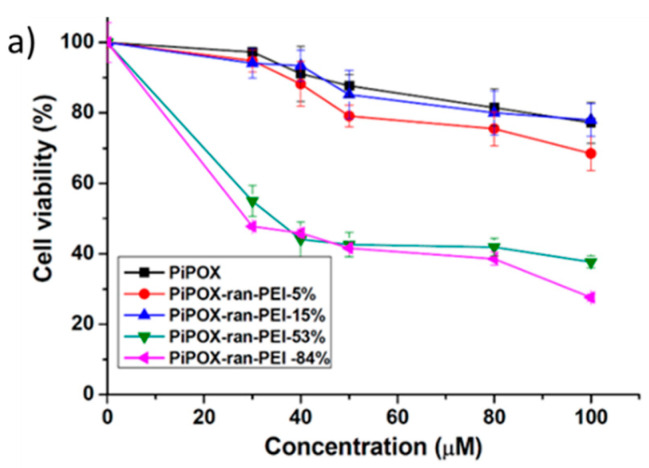

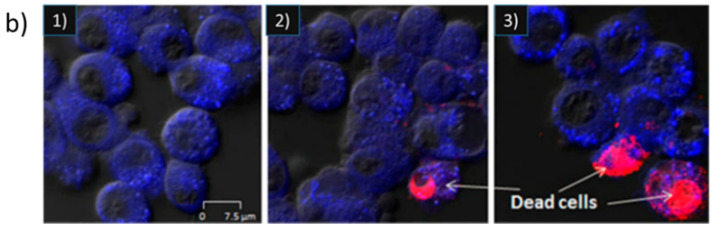
Cytotoxicity of POx–PEI copolymers. (**a**) Poly(iso-propyl-2-oxazoline) (PiPOx)–PEI copolymers differing by DH determined by MTT (3-(4,5-dimethyldiazol-2-yl)-2,5-diphenyltetrazolium bromide) dye reduction assay against the HEK 293 cell line. Adapted from Toncheva-Moncheva et al. as published by Elsevier [42]; (**b**) Confocal laser-scanning microscopy of macrophages P388.D1 treated with pure poly(2-ethyl-2-oxazoline) (PEtOx), PEtOx–PEI of 14, and 59% DH corresponding to images 1, 2 and 3, respectively. Adapted from Shah et al. as published by the Springer Nature [46].

It has been repeatedly shown that decreasing of DH substantially reduces the cytotoxicity of the copolymers, and this is relevant to all members of the POx family, including PMeOX [38], PEtOx [40,46,47,48,49], PiPOx [42], and PnPOx [41]. An example of the cytotoxicity evaluation of PiPOx–PEI copolymers against the non-tumorigenic cell line HEK 293 is shown in Figure 6a. The presented dose–response curves show a strong concentration-dependent cytotoxic effect accompanied by hard eradication provoked by the copolymers of higher DH (53–84%). A decrease of DH (<15%) caused a negligible inhibition of cellular viability even at higher concentrations [42]. The same was observed for other human cell lines with different cell types and origin –DOHH and EJ [42]. In a detailed in vitro study on the behavior of PEtOx–PEI copolymers of varying composition, Shah et al. [46] demonstrated an accumulation of copolymers in the macrophages with a minimal toxic effect. The cellular morphology remained typical for healthy cells, but the increase of DH initiated the presence of affected cells (Figure 6b). Fernandes et al. [47] studied the cytotoxicity of PEtOx–PEI copolymers of DP 100 by MTT (3-(4,5-dimethyldiazol-2-yl)-2,5-diphenyltetrazolium bromide) assay on a HeLa cell line. After 24 h of incubation, the copolymers of low DH did not induce any significant inhibition of cellular viability.

Interesting observations about the role of the alkyl substituents in the POx moieties have been made elsewhere [38,40,41,42]. Table 3 summarizes the results for the cell viability of selected POx–PEI copolymers characterized with approximately the same DP and DH. It is seen that the cytotoxicity of the POx–PEI copolymers is reduced by changing the alkyl side chain of the POx in the order *methyl > ethyl > iso-propyl > n-propyl*. The observed behavior could be directly related to POx hydrophobicity and water solubility, which decreases in the same order.

The DP has been shown to exert a well-pronounced effect on their ability to inhibit the proliferation and viability of cells [46,49,50,51]. Jeong et al. [49] found for PEtxO–PEI copolymers of nearly the same DH that the copolymer of higher DP exhibited stronger cytotoxic effects on NIH 3T3 fibroblast cells than that of the copolymer of lower DP. Similar effects have been observed by Shah et al. [46] on two more cell lines—macrophages P388.D1 and pancreatic bTC3 cells. The latter authors hypothesized that the lower cell viability of copolymers with a higher DP could be caused by a different distribution of positive charges along the polymer chains [46].

The copolymer chain topology is also a factor that can influence the biocompatibility of the polymers. Cook et al. [52] reported on hyperbranched PEtOx–PEI copolymers exhibiting lower toxicity compared to the linear counterpart. The authors attributed this to differences in the 3D structure and flexibility of the polymers, resulting in different interactions with the cellular surface [52]. Thus, the less flexible hyperbranched copolymer interacted with cell membranes with more difficulty than its more flexible linear equivalent and, hence, showed decreased toxicity [52]. A similar behavior was observed in a separate study [53] showing prominent dose-dependent cytotoxicity for a linear POx-based copolymer compared to the tested comb-like analogue. These authors also reported on the structural peculiarities of the comb-like copolymer topology in which PEI moieties were obstructed and shielded by POx segments.

## 4. Buffering Capacity of POx–PEI Copolymers

Along with the good biocompatibility, the copolymers should have the capacity to deliver and release DNA. The great advantage of the *gold standard* PEI is the presence of secondary and tertiary amino groups providing high buffering capacity [24,54]. Buffering capacity is defined as a measure of the ability of a material to resist changes in pH after the addition of acid or alkali. The high buffering capacity is associated with effective endolysosomal escape through the so-called *proton sponge* effect. The latter consists of osmotic swelling of the endolysosomes and their rupture [54], which is promoted by protonation of the polycationic vector and subsequent release of the polyplex and DNA in the cytoplasm. The escape from the endolysosomal pathway is of paramount importance to avoid the enzymatic degradation within the lysosomal compartment. 

The buffering capacity of POx–PEI copolymers has been evaluated by Jeong et al. [49]. The potentiometric titration of PEtOx–PEI copolymer solutions showed reduced buffering capacity compared to the control BPEI. The authors argued that a large fraction of amino groups of the PEI moieties remained unprotonated. In addition, inter- and intra-molecular hydrogen bonding between them and PEtOx carbonyl groups decreased the hydrogen exchange rate and thereby the protonation ability of the copolymers. In another study, the buffering capacity of hyperbranched PEtOx–PEI copolymers possessing only secondary amines has been determined [52]. BPEI and partially hydrolyzed LPEI of comparable DH were used as references. The results from the performed acid–base titration indicated a negligible effect of polymer architecture on the overall pKa value of POx–PEI copolymers. 

A strong composition-dependent buffering capacity was observed for a series of PMeOx–PEI copolymers differing in DP (50 and 200) and DH (37–99%) [38]. The authors presented the buffering capacity determined by standard acid–base titration as relative to that of BPEI, which was taken for 100% (Table 4). It was shown that the buffering capacity was strongly influenced by DH but only slightly influenced by DP. Nevertheless, all the investigated copolymers were shown to exhibit buffering capacity of over 50% that of BPEI, revealing their potential for endosomal escape.

## 5. Influence of POx–PEI Copolymer Characteristics and Temperature Effects on the Polyplexes Properties

In this section, we outline how parameters such as side chain substituents, DP, DH, and the chain topology of partially hydrolyzed POx influence their complexation ability and the physicochemical parameters of the resulting polyplexes. Special attention is paid to the effects of temperature on the properties of the polyplexes.

PMeOx–PEI copolymers have been reported to spontaneously form polyplex particles in water at ambient temperatures [38]. The easiness of formation was attributed to their high hydrophilicity, short alkyl side chain, and accessibility of the positive charges for complexation with DNA. The resulting polyplexes were well-defined, sub-100 nm in size, narrowly distributed (PDI < 0.05), and colloidally stable independently from the DP or DH of the copolymers. The enhanced colloidal stability of the systems was attributed to the PMeOX moieties located predominantly outwards. The polyplexes were invariably positively charged at higher (4–10) N/P ratios. Those based on PMeOx–PEI of a higher DP were characterized with more positive ζ potential (40 vs. 20 mV). 

Larger polyplex particles were obtained upon replacing PMeOx with PEtOx. In spite of the relatively high both DP (500) and DH (88%), Jeong et al. [49] reported a polyplex size of 150 nm at N/P = 60 and considerably larger at lower N/P ratios. The ζ potential was found to range in the 10–30 mV interval depending on the N/P ratio. Similar results have been reported by Bauer et al. [55] for a series of PEtOx–PEI copolymers of DP 200 and varying DH from 21% to 86%. The authors performed agarose gel electrophoresis, showing that all copolymers fully neutralized DNA at N/P 1–2 depending on the DH. However, only the polyplexes prepared at N/P ratios of 25 and 100, were found to effectively protect DNA from enzymatic degradation. The polyplex particles at these two N/P ratios ranged in size from 112 to 299 nm depending on the DH and exhibited strongly positive (19.5–47 mV) ζ potential.

**Figure 7:**
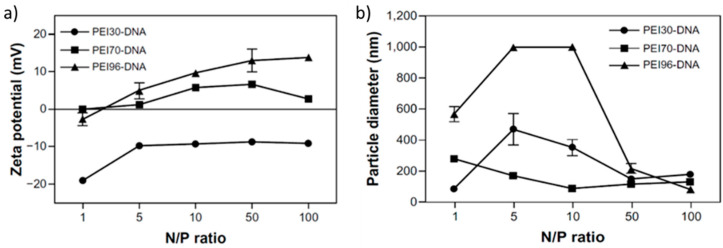
ζ potential (**a**) and size (**b**) variations of polyplex particles formed from PEtOx–PEI copolymers of DP 100. The copolymer concentration was 10 mg/mL. Adapted from Fernandes et al. as published by the Dovepress [47].

Fernandes et al. [47] investigated partially hydrolyzed (30–96%) PEtOx with a DP of 100. The binding ability was investigated by electrophoresis, demonstrating that full DNA neutralization was achieved at different N/P ratios depending on the DH. The size and ζ potential of the resulting polyplexes were determined in the interval of N/P ratios from 1 to 100. Throughout this range, PEtOx–PEI of lower DH (30%) formed invariably negatively charged particles, while those of higher DH exhibited a slightly positive ζ potential at N/P ≥ 5 (Figure 7a). The size variations were large and strongly dependent on the N/P and DH (Figure 7b).

The effects of DP and DH of a library of PEtOx–PEI copolymers have been studied by Blakney et al. [56]. The authors designed and synthesized copolymers with DPs ranging from 50 to 2000 with a variety of charge densities, which were represented by 20–100% DH. The polyplex size varied in the 80–400 nm range with a pronounced effect of DH (the higher the DH, the larger the size), while the effect of DP was not significant. In contrast, the ζ potential strongly depended on both DP and DH.

Bus et al. [57] described a strategy to introduce primary amine groups in the structure of PEtOx–PEI copolymers and reported on the superior performance of the resulting copolymers as gene delivery agents. The strategy consisted of the functionalization of the ethyleneimine units to introduce alkene groups, which were used to attach primary amine groups in the side chains by thiol-ene photo addition. The results indicated polyplex particles with pDNA that were smaller in size (diameter 143–158 nm vs. 242 nm), better defined (PDI 0.21–0.23 vs. 0.48), and more positively charged (ζ potential 23–27 mV vs. 20 mV) than those formed by the initial PEtOx–PEI copolymer.

**Figure 8:**
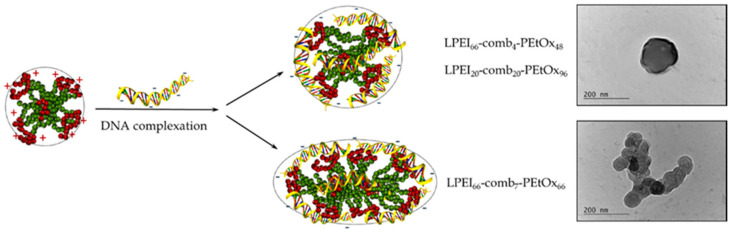
Schematic presentation of structure and morphology and TEM images of polyplex particles formed from PEtOx–PEI comb-like copolymers. Adapted from Haladjova et al. as published by the Wiley-VCH Verlag GmbH [40].

In the above examples, the temperature was not employed as a factor that can influence the properties and behavior of the copolymers and, hence, of the resulting polyplexes. As shown in the previous sections, favorable structural rearrangements take place upon heating of dilute copolymer dispersions above the LCST of the POx moieties. In a recent study, the complexion of PEtOx–PEI comb-like copolymers with DNA at elevated temperature has been explored [40]. Ethidium bromide quenching assay indicated the strong complexation ability of the comb-like copolymer particles with a pronounced effect of the PEI content. Small in size (R_h_ = 30–60 nm) polyplexes were observed in the whole range of N/P ratios (1–30), regardless of the copolymer structure and composition. All systems exhibited a strongly negative surface potential even at N/P ratios as high as 30. The authors argued that DNA was bound to the individual copolymer particles and was situated predominantly on their surface, as schematically presented in Figure 8. Detailed dynamic and static light scattering analyses revealed variations in polyplex morphology from spherical to elongated depending on the copolymer structure and composition. This was supported by transmission electron microscopy (TEM) where the existence of particles of a spherical and elongated shape was observed (Figure 8). 

**Figure 9:**
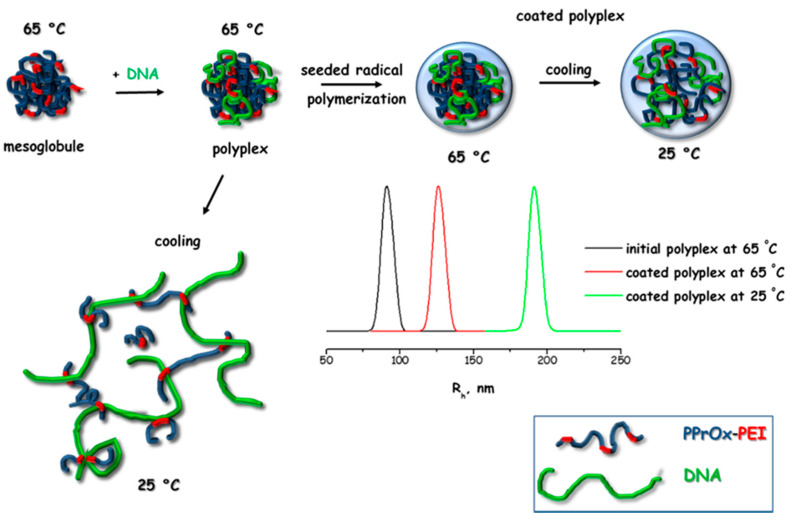
Schematic presentation of structural rearrangements of PnPOx–PEI polyplexes due to temperature variations and the proposed approach for their stabilization upon cooling. Adapted from Mees et al. as published by the American Chemical Society [41].

Internal structural rearrangements induced by changes in the solubility in water of the constituent moieties upon heating were found to have a strong effect on the DNA complexation in the case of thermoresponsive PPOx [41,42,43,53,58]. Thus, PnPOx–PEI copolymers of low DH (<10%) were unable to form complexes with DNA at low temperature [41]. However, upon heating at 65 °C positively charged mesoglobules composed of a large hydrophobic PnPOx core and a thin positively charged PEI shell were formed, which gave rise to the formation of well-defined polyplexes [41,53,58]. The complexes formed at 65 °C in the N/P ratio range from 0.5 to 10 were small in size, typically bellow 100 nm. The ζ potential variations with N/P followed a typical sigmoidal curve pattern, and the values turned from negative at a lower N/P to positive at a higher N/P. The DP and DH effects of the copolymers were negligible, while lowering the temperature below the LCST resulted in a significant increase of particle dimensions. This was related with PnPOx core disintegration and the transition to a molecularly dissolved state. However, DNA molecules remained bound to PEI moieties, acting as a physical cross-linking agent, which prevented the full disintegration of the polyplexes, as schematically presented in Figure 9. The resulting microgel particles were characterized with a nearly neutral ζ potential. Upon heating to physiological temperature, which is above the LCST of PnPOx, the initial structure of the polyplex particles was not restored. These unfavorable for biological application rearrangements were overcome by coating of the polyplex particles with cross-linked polymeric layer, which was prepared by the heterophase (seeded) radical copolymerization of N-isopropylacrylamide and N,N-methylenebis(acrylamide) (Figure 9), which acted as a physical barrier that provided mechanical strength and prevented strong swelling and disintegration of the initial particles upon cooling [41]. Thus, the initial structure and physicochemical characteristics of the systems were preserved [41].

The complexes of PiPOx–PEI (DH 5–84%) formed at elevated temperatures [42] were slightly larger than those of PnPOx–PEI, which was attributed to the more hydrophilic nature of PiPOx compared to PnPOx (see Figure 1 for the LCSTs of PiPOx and PnPOx). They were invariably negatively charged even at the highest N/P ratios, implying that DNA is situated on the outermost polyplex surface. Such an arrangement was favorable as DNA acted as a layer preventing enormous swelling of the polyplex particles upon cooling. Furthermore, the lowered solubility of PEI at lower temperatures counteracted the LCST properties of PiPOx, which prevented to a large extent the disintegration of the polyplexes. 

The copolymer chain topology has also been found to influence the complexation of POx–PEI copolymers with DNA [52,53]. The ability of hyperbranched and linear PEtOx–PEI copolymers to bind DNA was assessed by ethidium bromide quenching assay [52]. It was demonstrated that the hyperbranched copolymer displaced more effectively the dye, implying stronger complexation ability. The authors explained this behavior with different chain conformation states of the two copolymers and formation of more compact complexes in the case of the hyperbranched PEtOx–PEI copolymer, which was confirmed by DLS measurements and atomic force microsopy. In an earlier study [53], the physical properties of polyplexes based on linear and comb-like POx–PEI copolymers, formed at elevated temperatures, have been investigated. It was observed that the size of polyplexes based on the linear copolymer was independent from the N/P ratio. In contrast, those formed from the comb-like analogue were sensitive to N/P as their dimensions gradually decreased from micron- to nano-scale with increasing copolymer content. Lowering the temperature below LCST resulted in the instability of polyplexes based on the linear POx–PEI copolymer, while those based on the comb-like counterpart remained stable. The polyplexes of the comb-like copolymer were invariably negatively charged, in contrast to those of the linear copolymer, which exhibited positive ζ potential at N/P ≥ 2. Obviously, the diverse properties and behavior of the polyplexes reflected the different arrangements and structures of the polyplex particles arising from the different chain topology.

Summarizing this section, we may conclude that besides the DP and DH, other factors such as the substituent’s type and length, chain topology, and temperature can be identified to favorably influence the polyplex formation and properties. Increasing the length of the substituent in the order *methyl–ethyl–propyl* gradually deteriorates the polyplex formation. This has been attributed to lowering of the overall hydrophilicity of the copolymers as well as to the appearance of screening effects and steric hindrance. The non-linear chain topology is more preferable for the formation of well-defined polyplex particles of appropriate size and characterization parameters for cellular transfection. The formation of polyplexes at elevated temperatures has been recognized as appropriate particularly for copolymers bearing POx moieties exhibiting LCST properties because of the formation of particles with a favorable structure that serve as a platform for complexation with DNA.

## 6. Cellular Internalization and Transfection Efficiency of POx–PEI Gene Delivery Systems

In this section, the abilities of gene delivery systems based on POx–PEI copolymers to gain entry into cells and enable gene expression are evaluated and discussed.

The ability of PMeOx–PEI copolymers to introduce short PCR fragments (275 bp) or plasmid pUC19 (2686 bp) has been evaluated by SYBR Green staining [38]. Estimation by integrated fluorescence density was applied indicating that PMeOx–PEI successfully introduced both nucleic acids. Moreover, the PMeOx–PEI-based systems exhibited enhanced cellular penetration compared to the control BPEI, regardless of their DP and DH. This was attributed to the polyplexes’ small size taken together with the strong positive surface charge that were found to be beneficial for the endocytic uptake mechanism [9]. However, in spite of the effective cellular internalization, reduced transfection efficiency (less than 20%) was observed. To explain the results, the authors simulated endolysosomal conditions and investigated the behavior of the polyplexes. The findings revealed that PMeOx moieties restricted the internal repulsion between protonated amine groups responsible for the proton sponge effect, which retarded the swelling of polyplex particles and slowed down the endosomal rupture and DNA release. A summary of the macromolecular characteristics of the copolymers, physicochemical parameters of the polyplexes, and their transfection efficiency is presented in Table 5.

Jeong et al. [49] followed the influence of cationic charge density and molar mass on the transfection efficiency of PEtOx–PEI copolymers of DP of 500 and 2000. The DH, DP, and N/P ratio were found to have a crucial role in transfection against NIH 3T3 fibroblast. The authors showed that upon increasing the DH from 50% to 88%, the number of transfected cells significantly increased. Effective transfection was observed at high N/P ratios, and an N/P ratio of 25 appeared to be critical. A prominent DH dependence of transfection efficiency of PEtOx–PEI copolymers with a DP of 100 has been reported by Fernandes et al. [47]. The polyplexes formed from copolymers of DH of 70 and 96% at N/P ratios above 50 exhibited efficiency comparable to that of the control Lipofectamine. Due to the detected high toxicity of the PEtOx–PEI copolymer of 96% DH, only that of 70% DH was considered appropriate for gene delivery. The latter copolymer was characterized with lower toxicity due to the moderately positive ζ potential. The abilities of the same copolymers to interact with siRNA and to generate gene silencing were studied as well [47]. In comparison with DNA, the authors found that higher polymer to siRNA N/P ratios were required for binding, and the copolymers were less effective for application with siRNA. The common assumption that each copolymer works similarly well for each nucleic acid species has been questioned elsewhere [56]. These authors sought to optimize the DP (molar mass) and DH (charge density, PEI content) of a library of PEtOx–PEI copolymers for the formation of polyplexes with plasmid DNA, mRNA, and self-amplifying replicon RNA (repRNA). The optimal copolymer molar mass and charge density were found to be different for the different nucleic acid species, implying that the physical differences in the latter impacted how they interacted with cationic PEtOx–PEI copolymers [56]. Bauer at al. [55] reported for PEtOx–PEI copolymers with a DP of 200 that only the polyplexes, formed from copolymers with a DH > 75% at an N/P ratio of 25, showed efficiency comparable to the control LPEI and BPEI. According to the authors, the weaker DNA binding resulting in large in size polyplexes and the lower protective effect of PEtOx moieties against enzymatic degradation were the main reasons for poor transfection for the majority of the investigated systems. The gene delivery performance of linear PEtOx–PEI copolymers, which is consistent with that of the copolymers of the above studies, has been reported elsewhere [48,52,56]. The effects of macromolecular characteristics and physicochemical properties of the polyplexes on the transfection efficiency are summarized in Table 5. Apparently, good transfection efficiency, comparable to those of the control transfection agents, is achieved at high N/P (>20) ratios, DP (>150), and DH (>75%). 

The introduction of tertiary amines into the copolymer chain of PEtOx–PEI copolymers has been found to strongly influence the cellular uptake and transfection efficiency [57,59]. The polyplexes of the investigated terpolymers were characterized with fast and time-dependent cellular internalization via macropinocytosis. Effective transfection was observed at N/P ratios above 30. The terpolymers bearing tertiary amines [57] exhibited superior gene expression than the controls—linear PEI and related PEtOx–PEI copolymers with secondary amines only [59].

Haladjova et al. related the transfection efficiency of non-linear, comb-like PEtOx–PEI copolymers with the chain topology, shape of the polyplex particles, and internalization pathway [40]. The transfection efficiency did not exceed 35% relative to the control, but it should be noted that the PEI contents in this series of comb-like copolymers were below 40%, and the N/P ratio was as low as 8 (Table 5). The specificities of the copolymer composition and chain architecture (PEI main chain with different DPs and grafted PEtOx chains varying in number and length) as well as the temperature of preparation gave rise to the formation of polyplex particles of spherical or non-spherical morphologies. The authors speculated on the effects of the compactness of the polyplex particles and internalization pathway on the transfection efficiency. The negative ζ potential was also noteworthy (Table 5).

The transfection efficiency of PPOx–PEI copolymers has been poorly investigated. There is only one study for polyplexes of PnPOx–PEI copolymers [58]. The copolymers exhibited a remarkable transfection efficiency of 65%, which was detected on non-malignant HEK-293 and malignant REH cell lines compared to the control BPEI, considering both the low DH of the copolymer used (only 9%) and the low N/P ratio (N/P = 4). Upon coating the polyplex particles with a cross-linked polymer shell [41], which had a stabilizing effect, the transfection efficiency was reduced to 45% relative to the BPEI control (Table 5). The authors speculated that a longer time for more effective transfection was required for degradation of the polymer shell. Nevertheless, the good performance of the systems at, notably, low DH and N/P as well as their low toxicity makes them promising platforms for DNA delivery.

**Table 5:** Macromolecular characteristics, physicochemical properties, and transfection efficiency of polyplexes based on POx–PEI copolymers.

POx–PEI Copolymer	Molecular Chain Architecture	DP	DH, %	N/P Ratio	D_h_, nm	ζ Potential, mV	Cell Line	Time, h	Transfection Relative to Control, %	Reference
**PMeOX–PEI**	linear	50	41	6	60	27	H1299	24	20	[38]
			71		56	30			20	
		200	37		59	40			10	
			59		54	45			10	
**PEtOx–PEI**	linear	500	73	20	-	-	NIH 3T3	48	86	[49]
			88		300	20			98	
		2000	92		-	-			95	
	linear	100	81	20	246	46	HEK 293	24	80	[52]
	hyperbranched	172	32		499	5.4			15	
			58		65	22.3			30	
			76		53	24			80	
	comb-like	192	5	8	75	-30	PC3	24	25	[40]
		370	20		62	-38			35	
		404	40		112	-36			10	
**PnPOx–PEI**	linear	200	9	4	200 250 *	-	HEK 293, REH	24	65 45 *	[58]

* Data provided are for polyplexes coated with a cross-linked polymer shell. Tables may have a footer.

## 7. Final Remarks and Conclusions

POx–PEI copolymers are attractive materials for gene delivery, although they are presently somewhat under-researched and under-utilized. Combining POx moieties, exhibiting good biocompatibility, with DNA binding PEI moieties into a single copolymer chain is a promising approach to balance toxicity with transfection efficiency. Recent advances in the synthetic approaches, notably, the advent of the microwave reactors, the ease of preparation of POx (co)polymers of linear but also non-linear (star-like, hyperbranched) chain architecture as well as the controlled hydrolysis to introduce PEI segments in desired quantities and distribution allow precisely adjusting the efficiency-to-biocompatibility relationships, which is essential for the gene delivery vectors. 

The structural versatility of POx as well as the thermoresponsive LCST properties of some of them (PEtOx, PiPOx, and PnPOx) offers further advantages. The presence of different alkyl substituents influences the complexation ability of the POx–PEI copolymers, which, in turn, affects the physicochemical parameters of the resulting polyplexes. The highly water-soluble PMeOx–PEI interacts with DNA at ambient temperature, forming compact, small in size, and strongly positively charged polyplex particles. The complexes of the copolymers of PEtOx, the first thermosensitive member, are of a less compact structure, large in size, exhibiting nearly neutral or negative ζ potential even at higher N/P ratios. Therefore, very high N/P ratios (typically above 25) are needed to compact DNA and prepare polyplexes with optimal physicochemical parameters. The i-propyl and n-propyl substituents, which also introduce LCST properties in PPOx–PEI copolymers, strongly hinder the complexation with DNA at ambient temperatures. However, upon heating above their LCST, they form well-defined, positively charged mesoglobules—structures that are apparently appropriate for complexation with the oppositely charged nucleic acids. Indeed, the polyplexes formed at elevated temperatures are invariably of superior physicochemical characteristics and biological performance.

The alkyl substituents in the POx moieties reduce the cytotoxicity in the order *methyl > ethyl > i-propyl > n-propyl*. However, the polymerization degree and particularly the degree of hydrolysis have considerably stronger effects and might negate the effects of substituents. 

POx–PEI-based polyplexes successfully introduce DNA into the cells. The main reported internalization mechanism is endocytic uptake; however, alternative mechanisms have also been observed. The transfection efficiency of POx–PEI vectors is highly dependent on the DP, DH, and N/P ratio. Despite the good protonation ability of the copolymers, implying successful endolysosomal escape, comparable transfection efficiencies to that of control BPEI have typically been observed only at very high DP (>150), DH (>75%), and N/P (>25) ratios, which frequently compromise the biocompatibility of the systems. 

Without any doubts, the POx–PEI copolymers exhibit potential as non-viral vector systems. Their efficiency is typically lower than those of control PEI or other commercially available transfection agents, but this is counterbalanced by reduced toxicity and improved biocompatibility. The versatility of this class of copolymers in terms of the type of substituent, copolymer composition, degree of polymerization, degree of hydrolysis, and chain architecture, as well as the introduction of stimuli-responsive properties provide opportunities to finely tune the copolymer characteristics and physicochemical properties of the polyplexes to increase the biological performance. The results reported for the delivery of other nucleic acids (siRNA, mRNA, repRNA) are also encouraging. Further studies to provide insight into the structure and properties of these polyplexes, endosomal escape kinetics, in vitro and in vivo effectiveness, as well as the effects of external stimuli such as temperature are required.

## Figures and Tables

**Table 1:** Phase transition temperatures (T_CP_, °C) of PnPOx–PEI copolymers depending on the degree of hydrolysis (DH) (PEI content) and medium. The copolymer concentration was 10 mg/mL. The T_CP_ values were determined as the onset of aggregation from dynamic light scattering (DLS). Adapted from Mees et al. as published by the American Chemical Society [41].

% PEI	Water	BSA ^a^	pH 11	pH 5	KSCN ^b^
**0**	24.0	22.0	21.0	24.0	40.0
**3**	24.0	24.0	22.0	21.0	47.5
**5**	30.0	30.0	24.0	21.0	42.5
**11**	30.0	30.0	24.0	23.0	45.0
**16**	32.0	34.0	24.0	26.0	50.0
**35**	55.0	60.0	40.0	39.0	57.5
**58**	50.0	69.0	48.0	48.0	67.5
**65**	48.0	-	49.0	48.0	65.0

**^a^** bovine serum albumin concentration 0.3 M; **^b^** KSCN concentration 0.6 M.

**Table 2:** Variations of ζ potential of PnPOx–PEI copolymers with temperature and PEI content. The copolymer concentration was 10 mg/mL. Adapted from Mees et al. as published by the American Chemical Society [41].

Copolymer	ζ-Potential (mV)	
	25 °C	37 °C
**PPrOx_71_-PEI_4_**	−2.6 ± 5.3	25.5 ± 6.5
**PPrOx_68_-PEI_7_**	−6.3 ± 3.1	31.8 ± 8.4
**PPrOx_149_-PEI_11_**	−5.0 ± 4.4	19.6 ± 5.6
**PPrOx_146_-PEI_14_**	−6.1 ± 4.7	34.2 ± 6.7
**PPrOx_182_-PEI_18_**	−6.1 ± 4.2	19.0 ± 6.9

**Table 3:** Cell viability variations determined by MTT dye reduction assay for POx–PEI copolymers differing by their alkyl substituents. The copolymers are selected to be comparable by DP (≈70) and DH (≈15%).

Copolymer	Alkyl Residue	Cell Viability Taken at 30 mg/mL	Reference
PMeOx–PEI	methyl	92%	[38]
PEtOx–PEI	ethyl	81%	[40]
P*i*POX–PEI	*iso*-propyl	76%	[42]
P*n*POx–PEI	*n*-propyl	58%	[41]

**Table 4:** Buffering capacity of PMeOx–PEI copolymers differing by degree of polymerization (DP) (50 and 200) and DH (37–99%). The copolymer concentration was 0.5 mg/mL. a—the first and second digits correspond to DP and DH, respectively. b—the slope is determined from the titration curves. Adapted from Haladjova et al. as published by the Wiley Periodicals LLC [38].

Polymer ^a^	Buffering Capacity, ^b^ 1/slope	Buffering Capacity, % Relative to BPEI
**50-0**	27.6	19.0
**50-41**	72.0	49.6
**50-71**	94.3	65.0
**50-99**	137.9	95.0
**200-0**	26.8	18.5
**200-37**	75.6	52.1
**200-59**	94.8	65.3
**200-96**	139.9	94.5
**BPEI**	145.1	100.0

## References

[1] Miller D.A. (1992). Human gene therapy comes of age. Nature.

[2] Burton E., Glorioso J., Fink D. (2003). Gene therapy progress and prospects: Parkinson’s disease. Gene Ther..

[3] Cross D., Burmester J.K. (2006). Gene Therapy for Cancer Treatment: Past, Present and Future. Clin. Med. Res..

[4] Check E. (2002). Gene Therapy: A Tragic Setback. Nature.

[5] Jooss K., Chirmule N. (2003). Immunity to adenovirus and adeno-associated viral vectors: Implications for gene therapy. Gene Ther..

[6] Ratner M. (2012). First gene therapy approved. Nat. Biotechnol..

[7] Morrison C. (2015). $1-million price tag set for Glybera gene therapy. Nat. Biotechnol..

[8] Luo D., Saltzman W. (2000). Synthetic DNA Delivery Systems. Nat. Biotechnol..

[9] Jones C.H., Chen C.K., Ravikrishnan A., Rane S., Pfeifer B.A. (2013). Overcoming nonviral gene delivery barriers: Perspective and future. Mol. Pharm..

[10] Trentin D., Hubbell J., Hall H. (2005). Non-viral gene delivery for local and controlled DNA release. J. Control. Release.

[11] Al-Dosari M.S., Gao X. (2009). Nonviral gene delivery: Principle, limitations, and recent progress. AAPS J..

[12] O’Rorke S., Keeney M., Pandit A. (2010). Non-viral polyplexes: Scaffold mediated delivery for gene therapy. Prog. Polym. Sci..

[13] Pichon C., Billiet L., Midoux P. (2010). Chemical vectors for gene delivery: Uptake and intracellular trafficking. Curr. Opin. Biotech..

[14] Sunshine J.C., Bishop C.J., Green J.J. (2011). Advances in polymeric and inorganic vectors for nonviral nucleic acid delivery. Ther. Deliv..

[15] Tian H., Chen J., Chen X. (2013). Nanoparticles for Gene Delivery. Small.

[16] Wong S.Y., Pelet J.M., Putnam D. (2007). Polymer systems for gene delivery—Past, present, and future. Prog. Polym. Sci..

[17] Lungwitz U., Breunig M., Blunk T., Göpferich A. (2005). Polyethylenimine-based non-viral gene delivery systems. Eur. J. Pharm. Biopharm..

[18] Patnaik S., Gupta K.C. (2013). Novel polyethylenimine-derived nanoparticles for in vivo gene delivery. Expert Opin. Drug Deliv..

[19] Perche F., Benvegnu T., Berchel M., Lebegue L., Pichon C., Jaffres P.A., Midoux P. (2011). Enhancement of dendritic cells transfection in vivo and of vaccination against B16F10 melanoma with mannosylated histidylated lipopolyplexes loaded with tumor antigen messenger RNA. Nanomed. Nanotechnol. Biol. Med..

[20] Wang B., Zhang S., Cui S., Yang B., Zhao Y., Chen H., Hao X., Shen Q., Zhou J. (2012). Chitosan enhanced gene delivery of cationic liposome via non-covalent conjugation. Biotechnol. Lett..

[21] Nishiyama N., Kataoka K. (2006). Current state, achievements, and future prospects of polymeric micelles as nanocarriers for drug and gene delivery. Pharmacol. Ther..

[22] Xiong X.B., Falamarzian A., Garg S.M., Lavasanifar A. (2011). Engineering of amphiphilic block copolymers for polymeric micellar drug and gene delivery. J. Control. Release.

[23] Godbey W.T., Wu K.K., Mikos A.G. (1999). Poly(ethylenimine) and its role in gene delivery. J. Control. Release.

[24] Behr J.P. (1997). The Proton sponge: A trick to enter cells the viruses did not exploit. Chemia.

[25] Kafil V., Omidi Y. (2011). Cytotoxic impacts of linear and branched polyethylenimine nanostructures in a431 cells. Bioimpacts.

[26] Parhamifar L., Larsen A.K., Hunter A.C., Andresen T.L., Moghimi S.M. (2010). Polycation cytotoxicity: A delicate matter for nucleic acid therapy—Focus on polyethylenimine. Soft Matter.

[27] Adams N., Schubert U.S. (2007). Poly(2-oxazolines) in biological and biomedical application contexts. Adv. Drug Deliv. Rev..

[28] Hoogenboom R. (2009). Poly(2-oxazoline)s: A polymer class with numerous potential applications. Angew. Chem. Int. Ed..

[29] Schlaad H., Diehl C., Gress A., Meyer M., Demirel A.L., Nur Y., Bertin A. (2010). Poly(2-oxazoline)s as smart bioinspired polymers. Macromol. Rapid Commun..

[30] Luxenhofer R., Han Y., Schulz A., Tong J., He Z., Kabanov A.V., Jordan R. (2012). Poly(2-oxazoline)s as polymer therapeutics. Macromol. Rapid Commun..

[31] Brissault B., Kichler A., Guis C., Leborgne C., Danos O., Cheradame H. (2003). Synthesis of linear polyethylenimine derivatives for DNA transfection. Bioconjug. Chem..

[32] Lambermount-Thijs H.M., van der Woerdt F.S., Baumgaertel A., Bonami L., Du Prez F.E., Schubert U.S., Hoogenboom R. (2010). Linear poly(ethylene imine)s by acidic hydrolysis of poly(2-oxazoline)s: Kinetic screening, thermal properties, and temperature-induced solubility transitions. Macromolecules.

[33] Hoogenboom R., Thijs H.M., Jochems M.J., van Lankvelt B.M., Fijten M.W., Schubert U.S. (2008). Tuning the LCST of poly(2-oxazoline)s by varying composition and molecular weight: Alternatives to poly(N-isopropylacrylamide)?. Chem. Commun..

[34] Bludau H., Czapar A.E., Pitek A.S., Shukla S., Jordan R., Steinmetz N.F. (2017). POxylation as an alternative stealth coating for biomedical applications. Eur. Polym. J..

[35] Kakuda H., Okada T., Otsuta M., Katsumoto Y., Hasegawa T. (2009). Multivariate analysis of DSC–XRD simultaneous measurement data: A study of multistage crystalline structure changes in a linear poly(ethylene imine) thin film. Anal. Bioanal. Chem..

[36] Kakuda H., Okada M., Hasegawa T. (2009). Temperature-induced molecular structural changes of linear poly(ethylene imine) in water studied by mid-infrared and near-infrared spectroscopies. J. Phys. Chem. B.

[37] Kokufuta E., Suzuki H., Yoshida R., Yamada K., Hirata M., Kaneko F. (1998). Role of hydrogen bonding and hydrophobic interaction in the volume collapse of a poly(ethylenimine) gel. Langmuir.

[38] Haladjova E., Smolíček M., Ugrinova I., Momekova D., Shestakova P., Kroneková Z., Kronek J., Rangelov S. (2020). DNA delivery systems based on copolymers of poly(2-methyl-2-oxazoline) and polyethyleneimine: Effect of polyoxazoline moieties on the endo-lysosomal escape. J. Appl. Polym. Sci..

[39] Halacheva S., Price G.J., Garamus V.M. (2011). Effects of temperature and polymer composition upon the aqueous solution properties of comblike linear poly(ethylene imine)/poly(2-ethyl-2-oxazoline)-based polymers. Macromolecules.

[40] Haladjova E., Halacheva S., Momekova D., Moskova-Doumanova V., Topouzova-Hristova T., Mladenova K., Doumanov J., Petrova M., Rangelov S. (2018). Polyplex particles based on comb-like polyethylenimine/poly(2-ethyl-2-oxazoline) copolymers: Relating biological performance with morphology and structure. Macromol. Biosci..

[41] Mees M., Haladjova E., Momekova D., Momekov G., Shestakova P., Tsvetanov C., Hoogenboom R., Rangelov S. (2016). Partially hydrolyzed poly(n-propyl-2-oxazoline): Synthesis, aqueous solution properties, and preparation of gene delivery systems. Biomacromolecules.

[42] Toncheva-Moncheva N., Veleva-Kostadinova E., Tsvetanov C., Momekova D., Rangelov S. (2017). Preparation and properties of positively charged mesoglobules based on poly(2-isopropyl-2-oxazoline) and evaluation of their potential as carriers of polynucleotides. Polymer.

[43] Veleva-Kostadinova E., Toncheva-Moncheva N., Tsvetanov C., Rangelov S. (2017). Copolymers of 2-isopropyl-2-oxazoline and ethylenimine: Aqueous solution properties and polyplex formation. Nanosci. Nanotechnol..

[44] Meyer M., Antonietti M., Schlaad H. (2007). Unexpected thermal characteristics of aqueous solutions of poly(2-isopropyl-2-oxazoline). Soft Matter.

[45] Vlassi E., Pispas S. (2015). Solution behavior of hydrolyzed gradient methyl/phenyl oxazoline copolymers and complexation with DNA. Macromol. Chem. Phys..

[46] Shah R., Kronekova Z., Zahoranová A., Roller L., Saha N., Saha P., Kronek J. (2015). In vitro study of partially hydrolyzed poly(2-ethyl-2-oxazolines) as materials for biomedical applications. J. Mater. Sci. Mater. Med..

[47] Fernandes J.C., Qiu X., Winnik F.M., Benderdour M., Zhang X., Dai K., Shi Q. (2013). Linear polyethylenimine produced by partial acid hydrolysis of poly(2-ethyl-2-oxazoline) for DNA and siRNA delivery in vitro. Int. J. Nanomed..

[48] Hsiue G.H., Chiang H.Z., Wang C.H., Juang T.M. (2006). Nonviral gene carriers based on diblock copolymers of poly(2-ethyl-2-oxazoline) and linear polyethylenimine. Bioconjug. Chem..

[49] Jeong J.H., Song S.H., Lim D.W., Lee H., Park T.G. (2007). DNA transfection using linear poly(ethylenimine) prepared by controlled acid hydrolysis of poly(2-ethyl-2-oxazoline). J. Control. Release.

[50] Fischer D., Bieber T., Li Y., Elsässer H.P., Kissel T. (1999). A novel non-viral vector for DNA delivery based on low molecular weight, branched polyethylenimine: Effect of molecular weight on transfection efficiency and cytotoxicity. Pharm. Res..

[51] Kronek J., Kroneková Z., Lustoň J., Paulovičová E., Paulovičová L., Mendrek B. (2011). In vitro bio-immunological and cytotoxicity studies of poly(2-oxazolines). J. Mater. Sci. Mater. Med..

[52] Cook A., Peltier R., Zhang J., Gurnani P., Tanaka J., Burns J., Dallmann R., Hartlieb M., Perrier S. (2019). Hyperbranched poly(ethylenimine-co-oxazoline) by thiol–yne chemistry for non-viral gene delivery: Investigating the role of polymer architecture. Polym. Chem..

[53] Haladjova E., Rangelov S., Halacheva S., Mees M., Hoogenboom R., Momekova D. (2016). Influence of chain topology of poly(2-alkyl-2-oxazoline)-polyethyleneimine copolymers on DNA condensation. Nanosci. Nanotechnol..

[54] Nishikawa M., Huang L. (2001). Nonviral vectors in the new millennium: Delivery barriers in gene transfer. Hum. Gene Ther..

[55] Bauer M., Tauhardt L., Lambermont-Thijs H.M.L., Kempe K., Hoogenboom R., Schubert U.S., Fischer D. (2018). Rethinking the impact of the protonable amine density on cationic polymers for gene delivery: A comparative study of partially hydrolyzed poly(2-ethyl-2-oxazoline)s and linear poly(ethylene imine)s. Eur. J. Pharm. Biopharm..

[56] Blakney A.K., Yilmaz G., McKay P.F., Becer C.R., Shattock R.J. (2018). One size does not fit all: The effect of chain length and charge density of poly(ethylene imine) based copolymers on delivery of pDNA, mRNA, and RepRNA polyplexes. Biomacromolecules.

[57] Bus T., Englert C., Reifarth M., Borchers P., Hartlieb M., Vollrath A., Hoeppener S., Traeger A., Schubert U.S. (2017). 3rd generation poly(ethylene imine)s for gene delivery. J. Mater. Chem. B.

[58] Ivanova T., Haladjova E., Mees M., Momekova D., Rangelov S., Momekov G., Hoogenboom R. (2016). Characterization of polymer vector systems based on partially hydrolyzed polyoxazoline for gene transfection. Pharmacia.

[59] Hertz D., Leiske M.N., Wloka T., Traeger A., Hartlieb M., Kessels M.M., Schubert S., Qualmann B., Schubert U.S. (2018). Comparison of random and gradient amino functionalized poly(2-oxazoline)s: Can the transfection efficiency be tuned by the macromolecular structure?. J. Polym. Sci. Part A Polym. Chem..

